# Identifiying the ideal point of injection in ICG-assisted lymphatic-sparing Palomo procedure: A case report

**DOI:** 10.1016/j.eucr.2025.103269

**Published:** 2025-11-17

**Authors:** Giuseppe Autorino, Oana Sciboz, Oliver Sanchez, Enrico Broennimann

**Affiliations:** Centre Universitaire Romand de Chirurgie Pédiatrique, Division of Pediatric Surgery of Lausanne, CHUV, Lausanne, Switzerland

## Abstract

We report a 13-year-old boy with varicocele, testicular hypotrophy, and recurrent torsion-detorsion episodes who underwent combined ICG-assisted lymphatic-sparing laparoscopic Palomo varicocelectomy and bilateral orchidopexy. Three ICG injection sites were tested: (1) transcutaneous near the dartos of the spermatic bundle, (2) directly inside the dartos, and (3) beneath the parietal vaginalis tunica. Only (3) successfully identified lymphatic vessels adjacent to the spermatic vessels. The surgery was uneventful, and at 2 months postoperatively, pain resolved and varicocele improved clinically and sonographically. Injecting ICG into the parietal vaginalis tunica optimizes lymphatic visualization and may help standardize para-testicular ICG administration.

## 1 Introduction

Varicocele is a condition where altered venous drainage causes varices in the pampiniform plexus, affecting up to 15 % of males, particularly during puberty. Most varicoceles (95 %) are left-sided due to anatomical differences of the veinous drainage and the “nutcracker effect” of the superior mesenteric artery. This causes blood pooling around the testicle, potentially leading to pain, testicular hypoplasia, and negative impacts on semen parameters, sperm characteristics, testicular function, and fertility. However, only 20 % of men with varicoceles experience fertility issues, making treatment decisions in adolescents controversial.^1^^,^^2^ Diagnosis is mainly clinical, using palpation to detect dilated veins, with varicoceles graded on the Dubin-Amelar system. Scrotal color Doppler ultrasound is used to assess venous dilation, reflux, and testicular volume, influencing surgical decisions. Sarteschi's classification evaluates varicocele features and fertility impact.^3^ In adolescents, surrogate endpoints like testicular size and symptoms are used, with testicular hypotrophy and low volume correlating with reduced sperm counts. Treatment recommendations vary: 96 % of pediatric urologists recommend treating testicular hypotrophy, 79 % for testicular pain, and 39 % for altered semen parameters. It is not specified, however, the duration of testicular hypotrophy at the time of surgical treatment indication.^4^ Paternity studies show mixed results on the benefits of surgery.^2^^,^^5^ Surgical treatment for varicocele includes microsurgical ligation, sclerotherapy, and minimally invasive methods. Laparoscopic techniques, such as the Palomo and Ivanissevich procedures, ligate spermatic vessels, with variations sparing the artery but risking recurrence and atrophy. Both methods have a 10–30 % risk of post-operative hydrocele formation due to lymphatic damage.^5^ The first visualization of lymphatic vessels permitting a lymphatic-sparing technique, was achieved by injecting Methylene blue directly inside the testis,^6^ whereas the new fluorescent technology, using indocyanine green (ICG), has also enhanced visualization of lymphatics with minimal side effects when compared to methylene blue.^7^ In laparoscopic varicocelectomy, ICG helps reduce hydrocele risk by highlighting lymphatics, allowing for safer, more effective procedures for adolescents.^8^

## 2 Case presentation

A 13-year-old boy presented for evaluation following the incidental detection of a Grade III left-sided varicocele during a routine physical examination. While asymptomatic regarding the varicocele, he reported occasional episodes of sudden, severe scrotal pain, not always presenting on the left or right side, consistent with torsion-detorsion in the past. Sonography showed persistent significative asymmetry of testicular volume over 12 months (smaller left testicle, >20 %), with a grade IV varicocele according to Sarteschi's classification on the left testicle. In the absence of other explanation for the hypotrophy, surgical intervention was indicated to attempt to preserve testicular health and mitigate the risk of future torsion. ICG-guided lymphatic-sparing Laparoscopic Palomo varicocele ligation and bilateral orchidopexy were planned to address both conditions in a single operative session. Both the procedures were fully explained to the parents and the patient, that signed the informed consent.

### 2.1 Intervention

The procedure was performed under general anesthesia. The camera system was tested and the ICG was diluted with 5ml of sterile water, protected from the light until the operative team was ready to inject it. The first 10mm trocars was placed in a sub-umbilical access, then two other 5mm trocars were placed in the right iliac fossa and in the left flank. At this point, the parietal peritoneum was dissected and removed from the spermatic bundle. We also identified a left indirect hernia. 3 Once the preparation of the bundle was completed, the trans-scrotal orchidopexy began. After completing the right orchidopexy, the left testicle was exteriorized, and the camera was replaced in the abdomen before injecting the ICG. The first site of injection was through the skin near the spermatic bundle (Fig. 1). After the first injection, the lymphatics in the spermatic bundle did not uptake the fluorescence; however, the dye was evident medially to the spermatic bundle, under the parietal peritoneum (Fig. 2). The second injection was made directly inside the spermatic bundle, with similar results. In the end, ICG was injected inside the parietal vaginalis tunica (Fig. 3) and this allowed us to successfully visualize 2 lymphatic vessels inside the spermatic bundle (Fig. 4). The lymphatics were then spared and the dilated vessels were dissected with the LigaSure© one by one. Left orchidopexy was then completed without complications and the indirect hernia was treated laparoscopically. It was an uneventful day surgery procedure, with no intra-operative complications. At 1-week post-op follow-up he showed an infection on the right orchidopexy site that was completely treated with oral antibiotics. At 2 months post-op patient did not have any pain and a reduction of the varicocele both clinically and at the US was found. The right testicle had an estimated volume of 15.1 ml, compared to 14 ml in the pre-operative evaluation, within normal limits. The left testicle had an estimated volume of 13.6 ml, compared to 10 ml in the pre-operative evaluation, showing growth during time and reduction of the asymmetry (10 %).

**Picture 1:**
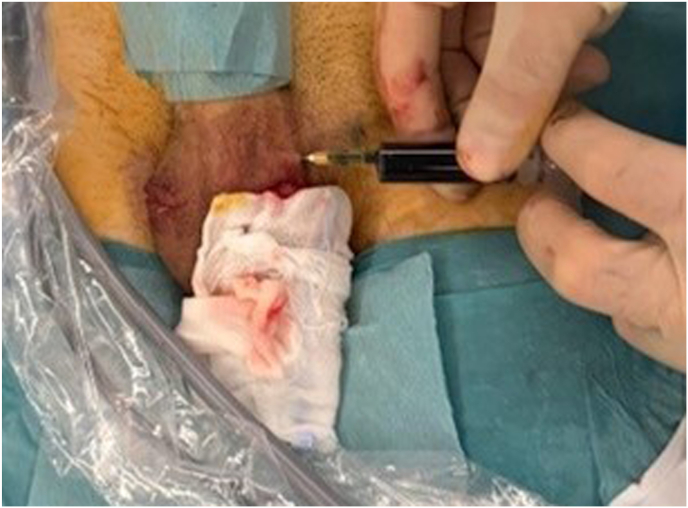
First site of injection.

**Picture 2:**
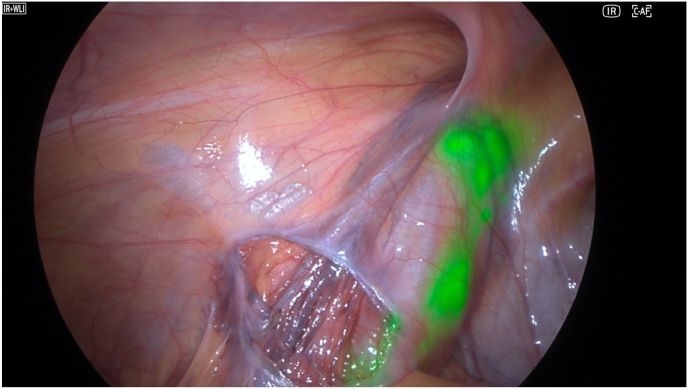
Extra-spermatic lymphatics.

**Picture 3:**
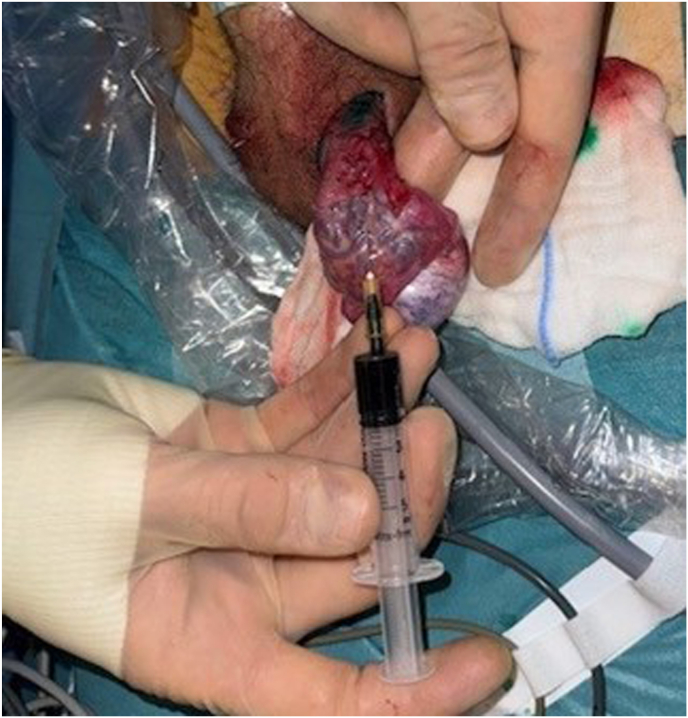
Third site of injection.

**Picture 4:**
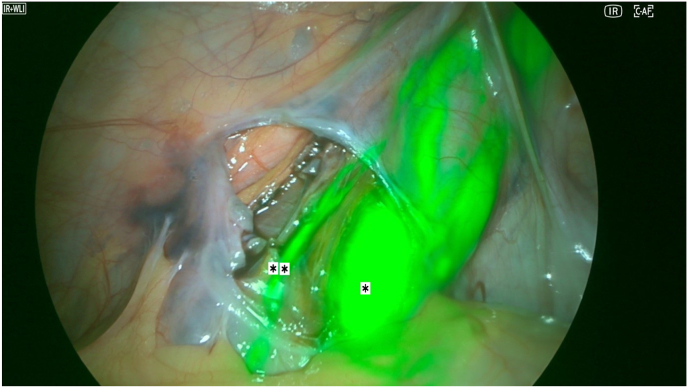
Extra-spermatic∗ lymphatics and intra-spermatic∗∗ lymphatics.

## 3 Discussion

The first attempt to identify and spare the lymphatics to avoid post-operative hydrocele through the use of a dye, was made employing methylene blue. As described in the experience of D'Alessio et al., the lymphatic mapping with methylene blue was not successful in all cases and it was associated with persistence of a blue spot at the injection site.^6^ Moreover, subsequent studies have shown that injection of methylene blue directly inside the testis could cause long-term testicular damage.^7^ Fluorescent technology, notably indocyanine green (ICG), has enhanced laparoscopic surgery by improving visualization of lymphatics and vessels. ICG fluoresces under near-infrared light, aiding identification of target structures with minimal side effects. It binds plasma 4 proteins after intravenous injection, with rapid hepatic uptake and biliary excretion. In laparoscopic varicocelectomy, ICG highlights lymphatics in all cases of intra-testicular injection, reducing hydrocele risk. Advanced cameras overlay fluorescence onto clear-light images, enabling precise identification and mapping of high-uptake areas. These techniques can provide safer, more effective surgical options for adolescents with varicoceles.^8^ Great controversy, however, is still present when debating the ideal site of injection for ICG. The most described method in literature is intra-testicular injection, with the injection of ICG performed with a 25G needle directly inside the testicular parenchyma. So far, this method has shown little to no complication in a mid-term follow-up.^9^ A recent study published by Zundel et al. has, however, shown some very interesting preliminary results on para-testicular injection, with a fluorescence reached in all 12 cases by injecting the ICG in three spots around the testicle, without the necessity to inject into the testicular parenchyma.^10^ By injecting at three para-testicular sites, however, the precise anatomical structure whose lymphatic vessels, running alongside the testicular vessels, have taken up the dye remains unclear. Potential candidates include the scrotal skin, the dartos, the parietal layer of the tunica vaginalis, or, through diffusion, the visceral layer of the tunica vaginalis or the tunica albuginea. We believe that with this case study we were able to provide a possible definitive answer to this question thanks to targeted injections into each individual structure, allowing real-time monitoring of intra-abdominal lymphatic staining. The need to perform a bilateral trans-scrotal orchidopexy and varicocele treatment in the same patient provided a unique opportunity to conduct a case study aimed at addressing this specific anatomical and physiological question.

## 4 Conclusion

ICG-guided Palomo procedure has risen to be a good alternative to treat varicocele in adolescents with little to no complications. Still, discussion exists on the ideal site of injection of fluorescent dye to reduce as much as possible potential damage to an already ill testicle. With the limits of a single case study, we believe that the concept of adsorption by the parietal vaginalis tunica could help create a new standard for injecting the dye in a para-testicular fashion, reducing the risk of iatrogenic testicular damage to a minimum. These findings will change our clinical practice, maybe introducing the use of intra-operative imaging to best direct the injection of the dye near the parietal vaginalis tunica.

## CRediT authorship contribution statement

**Giuseppe Autorino:** Writing – review & editing, Writing – original draft, Validation, Project administration, Methodology, Investigation, Data curation, Conceptualization. **Oana Sciboz:** Conceptualization. **Oliver Sanchez:** Conceptualization. **Enrico Broennimann:** Writing – review & editing, Writing – original draft, Validation, Supervision, Methodology, Data curation, Conceptualization.

## Declaration of competing interest

The authors declare that they have no conflicts of interest relevant to the content of this case report. No financial, personal, or professional relationships influenced the preparation and submission of this manuscript.

Generative AI and AI-assisted technologies were NOT used in the preparation of this work.
